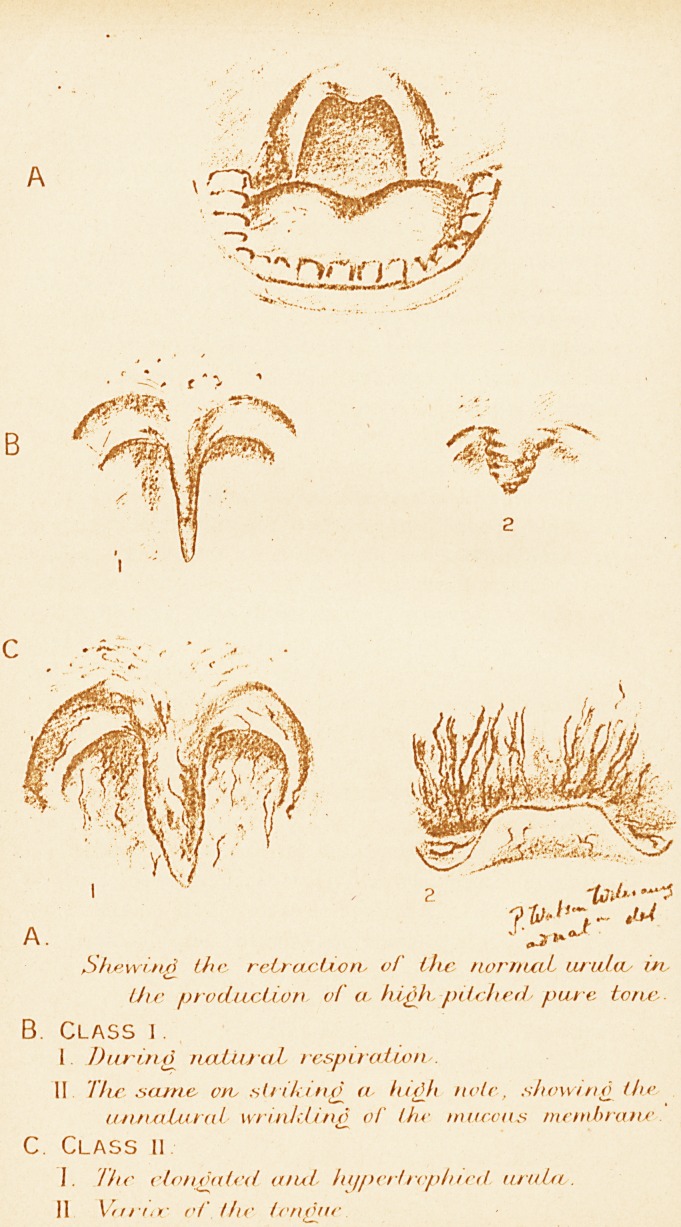# Chronic Pharyngitis with Elongated Uvula

**Published:** 1889-09

**Authors:** P. Watson Williams

**Affiliations:** Physician in charge of the Throat Department of the Bristol Royal Infirmary


### Clinical Records.

---

CHRONIC PHARYNGITIS WITH ELONGATED UVULA. By P. WATSON WILLIAMS, M.B. Lond., Physician in charge of the Throat Department of the Bristol Royal Infirmary.

Chronic Pharyngitis, or clergyman's sore-throat, is one of the most intractable conditions that are met with, and consequently has received much attention amongst laryngologists; but it is especially to those cases in which the *uvula* is relaxed and elongated that I invite attention in this paper.

The uvula is almost invariably more or less relaxed in chronic pharyngitis; and, while the elongation in some cases gives rise to no symptoms, it generally increases the unhealthy condition apart from any of the exciting causes which initiated the mischief, often producing alarming symptoms and requiring special treatment.

Labus of Milan very conveniently classifies the cases under two heads, viz:

Class I.—Those in which the uvula and soft palate are merely relaxed, otherwise remaining normal in appearance, there being no congestion or hypertrophy.

Class II.—Those in which there is hypertrophy and chronic congestion of the soft palate and fauces, often resulting in degeneration of the glandular structures of the naso-pharyngeal mucous membrane, and associated with severe constitutional disturbance. In these cases a varicose condition of the veins at the back of the tongue frequently results from constitutional irritation and prolonged congestion.

In the simpler cases, where there is merely relaxation of the uvula and soft palate without hypertrophy or congestion, the symptoms are mainly impairment of the quality and strength of the voice, and are chiefly observed in professional singers.

Labus, as the result of observation in 1,132 cases of professional singers who applied to him for treatment, found that the alteration and impairment of the voice was frequently due, not to the mere elongation of the uvula so much as to the paresis which resulted, preventing the proper and necessary movements of the uvula in singing high notes, and leading to forcing the voice, which becomes flat and tremulous and quickly tired. If the voice be still much used the constant strain may lead to chronic congestion and hypertrophy, and the case passes into the grave type. In a marked case the patient usually complains of continual hawking, with a feeling of some foreign body in the throat that cannot be coughed up, often likened to a hair or fish-bone in the throat. The cough may be very severe, especially at night on lying down, and on rising in the morning, often accompanied with nausea, and ending in vomiting from the tip of the uvula tickling the back of the tongue. Lennox Browne *(Diseases of the Throat)* mentions the case of a medical practitioner who consulted him, "complaining of constant pain in the left subscapular region, with irritable cough, loss of flesh, and impairment of general health. On the recommendation of two physicians, eminent in chest diseases, he sold his practice; but he entirely recovered his health after the removal of his uvula, and fourteen years later was still active and engaged in professional work."

A similar case, under my care at present, is rapidly recovering after removal of the uvula: this patient gained three pounds in weight in the first fortnight. Nor is it surprising that the loss of sleep and frequent vomiting should result in great emaciation and weakness, which, in association with cough, with expectoration of mucus streaked with blood from the pharynx, should lead one to suspect that the patient is suffering from tubercular disease of the lungs, especially in those who also complain of localised pains in the chest—pains which are purely reflex in origin.

The amount of blood that may be lost in this way is very great. One patient, now in the Bristol Royal Infirmary, had coughed up blood every morning for nine months, varying in amount from a teaspoonful to a cupful. His uvula has been removed, and he is rapidly recovering.

I recently saw a patient in consultation who suffered from severe and urgent attacks of dyspnœa on lying down at night, in addition to other minor symptoms, and thought to be due to some serious laryngeal affection. The dyspnœic attacks were the result of spasm of the glottis, caused by an elongated uvula; removal of the uvula prevented a recurrence of these alarming attacks.

A variety of causes may lead to this elongation of the uvula, the commonest cause being the using of the voice during catarrhal attacks—a time when all the tissues of the naso-pharynx are congested and weakened; it is therefore most frequently found in those with whom speaking or singing is a means of livelihood or profession, such as clergymen, schoolmasters, actors, etc.

The paresis of the soft palate is probably due to peripheral neuritis of the posterior palatine nerve from compression at its exit from the posterior palatine canal, containing the motor nerve fibres for the levator palati and azygos uvulæ, as well as the sensory nerve supply for the velum palati, and being exactly analogous to Bell's paralysis due to cold, and resulting from compression of the facial nerve at its exit from the stylo-mastoid foramen. Woakes points out that this implication of the posterior palatine nerve accounts for the paresis of the soft palate so frequently associated with "necrosing ethmoiditis."

Another frequent cause is nasal obstruction, causing the patient to breathe through the mouth. Minor degrees of nasal obstruction are frequently overlooked; but compelling the patient to sleep with the mouth open, he wakes in the morning with a dry parched tongue. It is not surprising that slight, but oft repeated, attacks of naso-pharyngeal congestion should result from this unnatural respiration.

Acute pharyngitis and other predisposing conditions are numerous: such as chronic portal congestion, chronic gastric catarrh, alcoholism, the tobacco-habit, phthisis and chronic bronchitis, breathing in crowded rooms and dusty atmospheres.

In treating cases of relaxed uvula, it is well to give local astringent applications a fair trial, especially in the milder cases. These must be applied to the back of the soft palate and the posterior nares, as well as to the fauces.

In one case, in a child, where local astringents failed, and removal of the elongated uvula was objected to, I obtained a cure by daily local Faradisation for three weeks. I need hardly add that this is an unnecessarily tedious and expensive process.

A

B

1

2

C

1

2

P. Watson Williams ad nat. del

A.

*Shewing the retraction of the normal uvula in the production of a high-pitched pure tone.*

B. Class I.

I. *During natural respiration.*

II. *The same on striking a high note, showing the unnatural wrinkling of the mucous membrane.*

C. Class II.

I. *The elongated and hypertrophied uvula.*

II. *Varix of the tongue.*

When local applications have failed, the uvula ought certainly to be partially removed. In the severer cases it is better to do this little operation at once. In properly selected cases there is no simple operation which is more urgently called for, and which brings about such excellent results in so short a time.

Cases of elongated uvula, pure simple relaxation, are not always easily recognised. In doubtful cases the patient should be directed to open the mouth and breathe quietly. At first the uvula will be partially retracted into the soft palate, but, if "elongated," it soon drops, and the tip rests on the back of the tongue. On striking a high note, the normal uvula is almost completely drawn up in to the soft palate, which is raised, but the relaxed uvula is shortened in wrinkles of redundant mucous membrane (*vide* Figure B2.) The redundant translucent mucous membrane is obvious along the free edges of the velum and at the tip of the uvula.

I have had a large number of cases, and have always obtained good results by ablation of the uvula. Labus, as the result of his extensive experience in ablation of the uvula in professional singers, was convinced that when carefully done, so as only to remove the abnormally elongated portion of the uvula, the operation produced no deterioration, but almost invariably a marked improvement, in the quality of the voice.

In all cases the nasal passages should be examined for obstruction. If obstruction exists, it must be appropriately treated if a good result is to be obtained and a permanent cure effected.

## Figures and Tables

**A. B. C. f1:**